# Evaluating the impact of Sacubitril/valsartan on diastolic function in patients with heart failure: A systematic review and meta-analysis

**DOI:** 10.1097/MD.0000000000037965

**Published:** 2024-05-10

**Authors:** Jinfu Li, Yanbin Song, Fengyun Chen

**Affiliations:** a Department of Internal Medicine III, Fujian Dehua County Hospital, Quanzhou, Fujian Province, China; b Department of Cardiology, Wujin Hospital Affiliated with Jiangsu University, Changzhou, Jiangsu, China; c Department of Cardiology, the Wujin Clinical College of Xuzhou Medical University, Changzhou, Jiangsu, China; d Department of Cardiovascular Medicine, Central Hospital Affiliated to Chongqing University of Technology, Chongqing, China.

**Keywords:** diastolic dysfunction, heart failure, meta-analysis, Sacubitril/valsartan

## Abstract

**Background:**

Heart failure is a common and severe condition, often complicated by diastolic dysfunction. Current standard therapies such as ACEIs and ARBs have limited efficacy in managing diastolic function. Sacubitril/Valsartan, an emerging therapy, warrants rigorous investigation to elucidate its impact on diastolic function in heart failure patients.

**Methods:**

This systematic review and meta-analysis were conducted adhering to Preferred Reporting Items for Systematic Reviews and Meta-Analyses guidelines and utilized the PICO schema. Searches were performed on 4 databases—PubMed, Embase, Web of Science, and Cochrane Library—without temporal restrictions. Inclusion and exclusion criteria were strictly defined, and quality assessments were conducted using the Cochrane Collaboration Risk of Bias tool. Both fixed-effects and random-effects models were used for statistical analysis, depending on inter-study heterogeneity assessed by *I*^2^ statistics and Chi-square tests.

**Results:**

Out of 1129 identified publications, 8 studies met the criteria and were included in the meta-analysis. These studies consisted of both randomized controlled trials and cohort studies and featured diverse global populations. Significant reductions were found in the echocardiographic parameter E/e’ ratio and LAVi upon treatment with Sacubitril/Valsartan compared to standard therapies, with mean differences of −1.38 and −4.62, respectively, both with *P* values < .01.

**Conclusions:**

This meta-analysis demonstrates that Sacubitril/Valsartan significantly improves diastolic function parameters in heart failure patients compared to standard treatments. These findings underscore the potential benefits of Sacubitril/Valsartan in the management of heart failure, particularly for patients with diastolic dysfunction.

## 1. Introduction

Heart failure (HF) is a burgeoning public health crisis, accounting for significant morbidity, mortality, and financial burden on healthcare systems globally.^[1]^ This complex clinical syndrome arises from multiple etiological factors and manifests through a range of symptoms and complications, underlining its multifaceted nature. Among the various pathological mechanisms contributing to the onset and progression of HF, diastolic dysfunction has been spotlighted as a pivotal component, especially in the subset of patients categorized with heart failure with preserved ejection fraction (HFpEF).^[2]^ Diastolic dysfunction of the left ventricle leads to elevated intraventricular pressures during cardiac relaxation, a crucial pathophysiological event. The ensuing abnormal hemodynamics can catalyze the onset of pulmonary arterial hypertension and systemic venous congestion, thereby precipitating a detrimental cycle that aggravates the HF condition.^[3,4]^ This deleterious cascade elucidates the dire need for effective therapies targeting diastolic dysfunction, a realm that current pharmacological interventions have not sufficiently addressed.

Historically, angiotensin-converting enzyme inhibitors (ACEIs) and angiotensin II receptor blockers (ARBs) have been the cornerstone of HF treatment.^[5]^ Yet, these classes of drugs have shown inadequate effectiveness in tackling the root issue of diastolic dysfunction, leaving a considerable therapeutic void. In 2015, the pharmacological landscape for HF was revolutionized by the introduction of Sacubitril/Valsartan (commercial name LZ969), an angiotensin receptor-neprilysin inhibitor.^[6]^ Clinical trials conclusively demonstrated its efficacy in reducing all-cause mortality and the rate of HF-related hospitalizations, outperforming traditional ACEIs like enalapril.^[7]^ Moreover, Sacubitril/Valsartan has shown particular promise in attenuating myocardial remodeling, a phenomenon strongly correlated with HF progression.

Notwithstanding its clinically verified benefits, the impact of Sacubitril/Valsartan on diastolic function remains a subject of ongoing debate. While some preliminary investigations suggest potential benefits, conclusive evidence remains elusive. This discernible lack of consensus necessitates a methodical inquiry into the matter. Therefore, this systematic review and meta-analysis aim to elucidate the efficacy of Sacubitril/Valsartan in comparison to conventional therapeutic agents for the improvement of diastolic function in HF patients. By synthesizing the available data, this study aims to provide a new evidence-based foundation for the management of diastolic dysfunction in HF, thereby filling the existing gaps in our understanding and aiding in the refinement of treatment guidelines.

## 2. Materials and methods

### 2.1. Search strategy

In executing this meta-analysis and subsequently disseminating our findings, we strictly complied with the guidelines delineated by the Preferred Reporting Items for Systematic Reviews and Meta-Analyses.^[8]^ The structure of the meta-analysis was based on the PICO (Patient, Intervention, Comparison, Outcome) schema, clarifying the following domains: Patient (P): The population of interest consisted of adult patients diagnosed with HF, with a particular focus on those experiencing diastolic dysfunction. Intervention (I): The intervention examined was the administration of Sacubitril/Valsartan in treating HF. Comparison (C): The comparison group included patients treated with standard therapies for HF, such as ACEIs or ARBs. Outcome (O): The primary outcomes measured were the changes in diastolic function parameters and secondary outcomes included all-cause mortality and rates of HF-related hospitalizations.

Four academically recognized electronic databases—PubMed, Embase, Web of Science, and the Cochrane Library—were systematically queried on July 19, 2023, without any temporal restrictions imposed. The search algorithm was constructed using a select group of keywords, including “heart failure,” “diastolic dysfunction,” “Sacubitril/valsartan,” “angiotensin-converting enzyme inhibitors,” and “angiotensin II receptor blockers.” These terms were deliberately chosen to align with the comprehensive reach of the PICO model and to guarantee an exhaustive retrieval of pertinent research articles for the meta-analysis. There were no linguistic constraints enforced, and bibliographies of germane publications were manually inspected to identify any supplementary applicable sources.

In detail, our search strategy involved using the following query string, tailored for each database to accommodate its specific indexing and search capabilities: PubMed: (“heart failure”[MeSH Terms] OR “heart failure”[All Fields]) AND (“diastolic dysfunction”[MeSH Terms] OR “diastolic dysfunction”[All Fields]) AND (“Sacubitril/valsartan”[MeSH Terms] OR “Sacubitril/valsartan”[All Fields] OR “LCZ696”[All Fields]) AND (“angiotensin-converting enzyme inhibitors”[MeSH Terms] OR “ACEIs”[All Fields] OR “angiotensin II receptor blockers”[MeSH Terms] OR “ARBs”[All Fields]). Embase: “heart failure”/exp OR “heart failure” AND “diastolic dysfunction”/exp OR “diastolic dysfunction” AND “Sacubitril valsartan”:ti,ab,kw OR “LCZ696”:ti,ab,kw AND “angiotensin-converting enzyme inhibitors”/exp OR “ACEIs” OR “angiotensin receptor antagonists”/exp OR “ARBs.” Web of Science and Cochrane Library were searched using a similar strategy, appropriately adjusted for the syntax supported by each database. This comprehensive search was designed to capture all relevant studies on the impact of Sacubitril/Valsartan on diastolic function in HF patients, without restrictions on language or publication date.

### 2.2. Inclusion criteria and exclusion criteria

For the systematic review and meta-analysis, studies were required to fulfill the following inclusion criteria: The study population needed to consist of patients diagnosed with HF; Interventions in the study had to include standard treatment with Sacubitril/Valsartan; There were restrictions on the types of study designs; randomized controlled trials (RCTs), cohort studies; Studies needed to report at least one of the predetermined outcome measures.

Conversely, the following were the exclusion criteria: Studies that were published multiple times were excluded; Manuscripts with incomplete, unclear, or inconsistent analytical data and outcome measures were eliminated; Studies deemed of poor quality or those lacking original data were not considered; Contributions in the form of case reports, expert opinions, and narrative reviews were excluded from the analysis.

### 2.3. Data extraction

Data extraction for the meta-analysis was executed independently by 2 reviewers, followed by a cross-verification process to ensure accuracy. In instances where discrepancies arose, the involved reviewers engaged in discussion to resolve these inconsistencies. Consultation with a third reviewer was considered if a resolution could not be reached. Diastolic function was assessed using key indicators such as the ratio of the early mitral inflow velocity to the early diastolic mitral annular velocity (E/e’), the ratio of early to late mitral inflow peak velocities (E/A), and the left atrial volume index (LAVi). Specific data extracted from the studies comprised the author name, study type, geographic region of the study, target population, gender ratio, sample size, duration of follow-up, intervention methods, and primary outcome measures including changes in E/e’, E/A, and LAVi. For studies that did not provide changes in these parameters, pre- and post-treatment data were extracted. If the required data were not available in the published material, we initiated contact with the original investigators via email to request unpublished data. This data extraction procedure aligns with the standardized practices typically employed in the conduct of a systematic review and meta-analysis.

### 2.4. Quality assessment

The integrity of the studies incorporated into the meta-analysis was rigorously appraised utilizing the Cochrane Collaboration Risk of Bias assessment tool (version ROB 1.0).^[9]^ Two independent assessors critically examined the studies across multiple domains, which included the randomization process, concealment of allocation, the blinding of participants and investigators, completeness of outcome data, selective outcome reporting, and any additional sources that could introduce bias. Each of these domains was categorized as either posing a low, unclear, or high risk of bias. In instances where the independent assessments diverged, consensus was reached through a deliberative process, and if needed, consultation with a third evaluator was invoked to resolve the disagreement.

### 2.5. Statistical analyses

Inter-study heterogeneity was evaluated through the Chi-square test and quantitatively gauged using the *I*^2^ statistic. A lack of substantial heterogeneity was implied if the *I*^2^ index fell below 50% and the corresponding *P* value was equal to or exceeded .10. In these instances, the fixed-effects model was applied to estimate the aggregated effect size. Conversely, an *I*^2^ value at or above 50%, or a *P* value under .10, signaled the presence of significant heterogeneity, prompting the utilization of the random-effects model for estimating the aggregated effect size. To explore the underpinnings of such heterogeneity, sensitivity analyses were undertaken. These involved a systematic exclusion of each individual study, followed by a recalculation of the collective effect size to assess the stability and resilience of the findings. To identify the likelihood of publication bias influencing the meta-analysis, funnel plot symmetry was scrutinized. A balanced distribution of data points around the funnel plot apex indicated a diminished probability of result skewness due to publication bias. For a quantitative assessment of potential publication bias, Egger linear regression test was executed. All statistical inferences were drawn from 2-tailed tests, considering a *P* value <.05 as indicative of statistical significance. Analyses were conducted using Stata software, version 17 (StataCorp, College Station, TX).

## 3. Results

### 3.1. Search results and study selection

**Figure 1.**
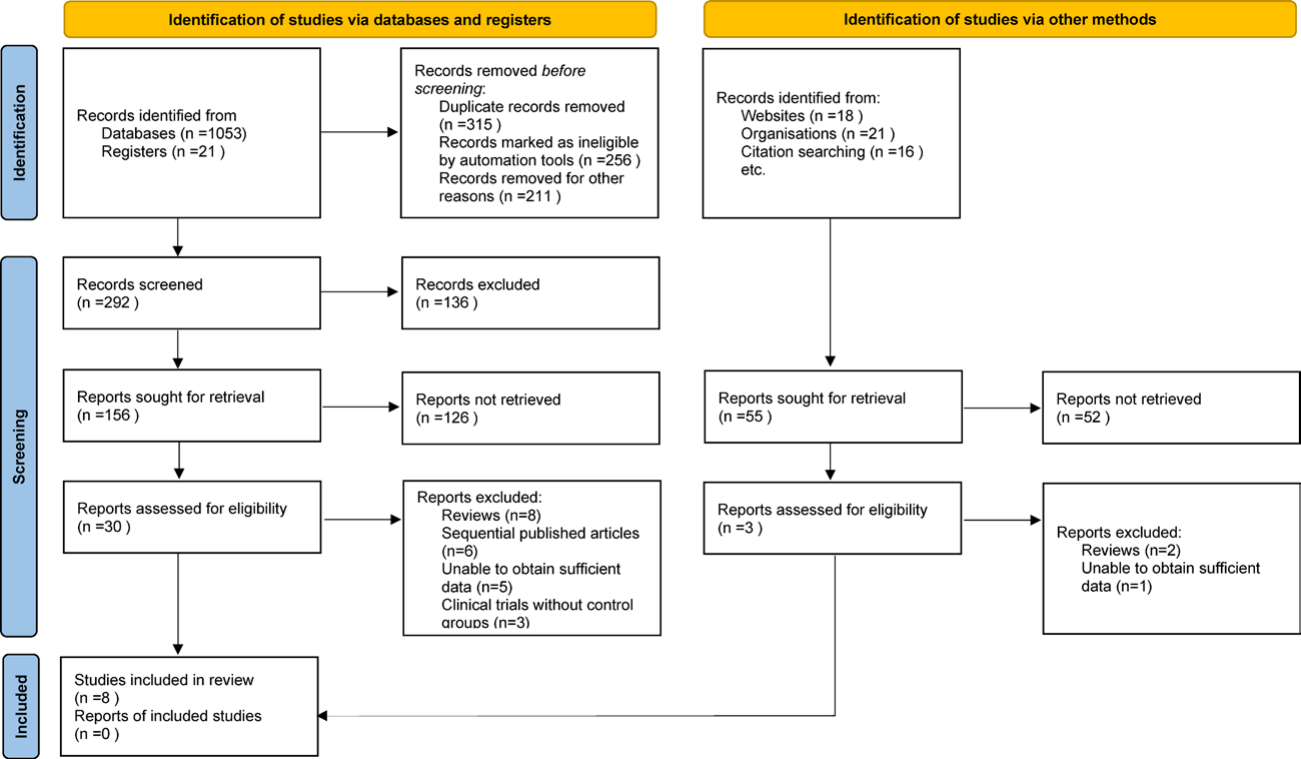
Selection process of included studies.

In the preliminary database search, a total of 1129 relevant publications were identified. Following the removal of duplicate entries and a meticulous review of titles and abstracts in accordance with our pre-defined inclusion and exclusion criteria, 30 pertinent articles were shortlisted. Subsequent to further full-text evaluation, 22 of these were excluded, culminating in the inclusion of 8 studies in the meta-analysis.^[10–17]^ The schematic representation of this literature selection process and its outcomes can be viewed in Figure 1.

### 3.2. Study characteristics

In this meta-analysis, the study characteristics span a variety of study designs, including RCTs and Prospective and Retrospective Cohort studies, conducted from 2012 to 2021. The studies are globally diverse, hailing from the United States, Brazil, Egypt, South Korea, Greece, China, and a multi-country collaboration. The age of the cohorts ranged from approximately 56 to 68 years, with varying percentages of female participants. These studies predominantly focus on Heart Failure with preserved Ejection Fraction (HFpEF), except for one that investigates HFpEF. Control groups largely received enalapril, Valsartan, or ACEI/ARB therapies. Sample sizes varied significantly, ranging from 44 to 464 in the experimental groups. Ejection fractions were generally ≤40%, with baseline measures indicating considerable cardiac impairment. Follow-up durations extended from 3 to 36 months (Table 1).

**Table 1:** Characteristics of studies included in the meta-analysis.

First Author	Yr of Publication	Country	Study type	Age (yr)	Female (%)	Control group	Sample size (experimental group)	Target population	Ejection fraction (%)	Baseline EF (%)	Follow-up duration (mo)
Desai^[10]^	2019	United States	RCT	67.3 ± 9.1	109 (23.5)	Enalapril	464 (231)	HFrEF	≤40	34 ± 10	12
Dos Santos^[11]^	2021	Brazil	RCT	56.4 ± 10.2	13 (29.5)	Enalapril	44 (26)	HFrEF	<40	33 ± 10	24
Elshafey^[12]^	2021	Egypt	Prospective Cohort	61.0 ± 9.1	19 (39.6)	Standard HF Therapy	73 (36)	HFrEF	≤40	27.07 ± 7.06	6
Kang^[13]^	2019	South Korea	RCT	68.0 ± 10.0	46 (39.0)	Valsartan	118 (60)	HFrEF	25~50	26.08 ± 12.07	12
Miric^[14]^	2021	United States	RCT	62.6 ± 11.2	16 (23.5)	ACEI/ARB	68 (34)	ADHF	≤40	32.51 ± 5.65	3
Nakou^[15]^	2018	Greece	Prospective Cohort	61.5 ± 9.7	19 (39.6)	Valsartan	48 (23)	HFrEF	≤35	33.3 ± 7.2	6
Solomon^[16]^	2012	14 Countries	RCT	63.3 ± 13.9	170 (56.5)	Valsartan	301 (149)	HFpEF	≥45	34.3 ± 7.1	36
Sun^[17]^	2021	China	Retrospective Cohort	64.1 ± 13.9	101 (30.0)	ACEI/ARB	336 (168)	HFrEF	<40	32.8 ± 7.2	19.4

14 Countries include: Argentina, Brazil, Canada, Germany, India, Italy, the Netherlands, Poland, Romania, Russian Federation, Singapore, Spain, United States, Venezuela.

ACEI = Angiotensin-Converting Enzyme Inhibitors, ADHF = Acute Decompensated Heart Failure, ARB = Angiotensin II Receptor Blockers, HFpEF = Heart Failure with Preserved Ejection Fraction, HFrEF = Heart Failure with Reduced Ejection Fraction, RCT = Randomized Controlled Trial.

### 3.3. Results of quality assessment

**Figure 2.**
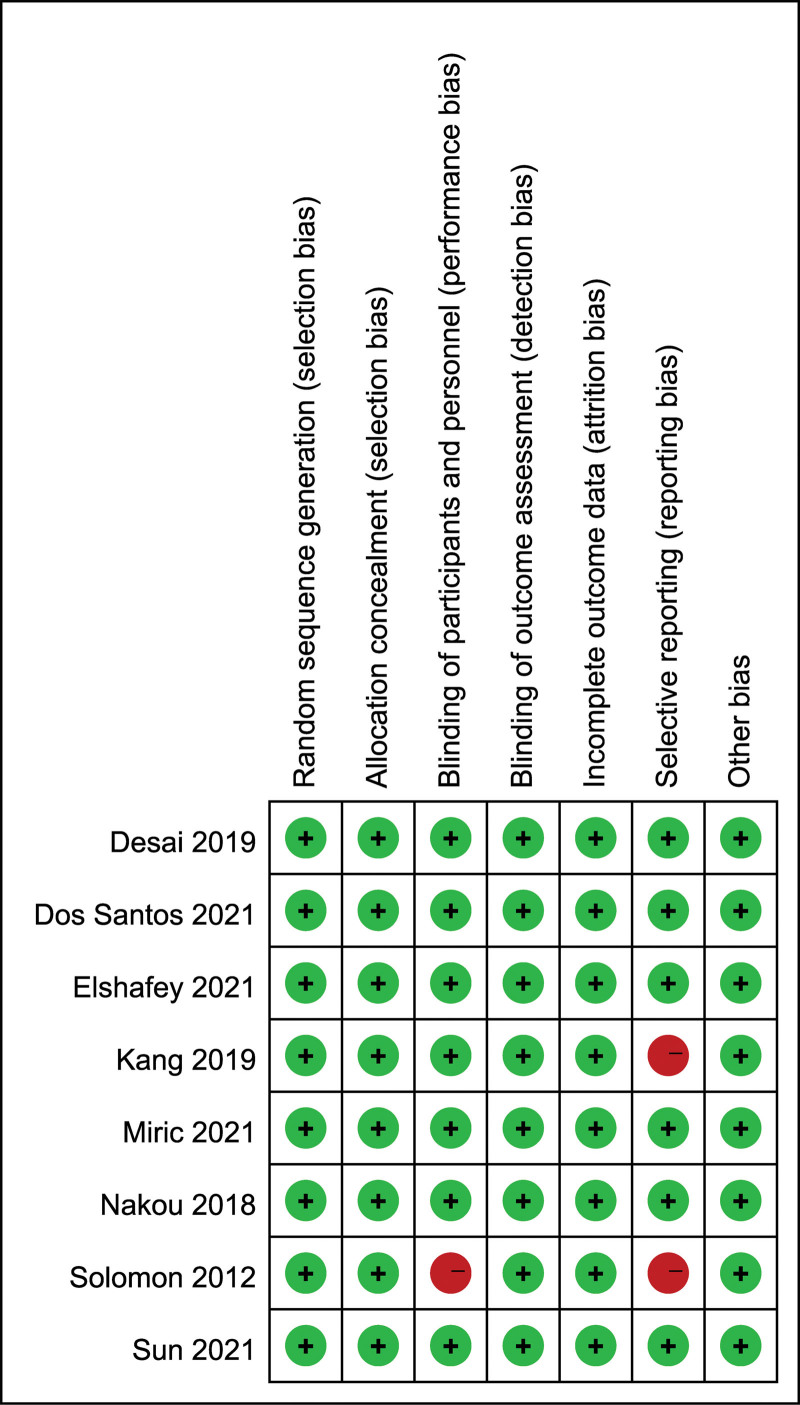
Quality assessment of included studies using Cochrane Collaboration tool criteria. Red in figure indicates high risk, yellow represents unclear risk and green means low risk.

In the meta-analysis, risk of bias was systematically assessed across multiple facets in the 8 incorporated studies. Of these, half exhibited low susceptibility to bias across all evaluative dimensions, signifying robust methodological quality. Nonetheless, one-eighth of the examined studies manifested a heightened risk in the arena of participant and staff blinding, raising concerns about the introduction of performance bias that could potentially skew the study outcomes. Additionally, a quarter of the RCTs under review displayed elevated risks associated with selective outcome reporting, inferring that the integrity of the overall study results could be compromised by incomplete or biased dissemination of findings (Fig. 2).

### 3.4. Meta-analysis on impact of Sacubitril/valsartan on early mitral inflow velocity to early diastolic mitral annular velocity ratio

**Figure 3.**
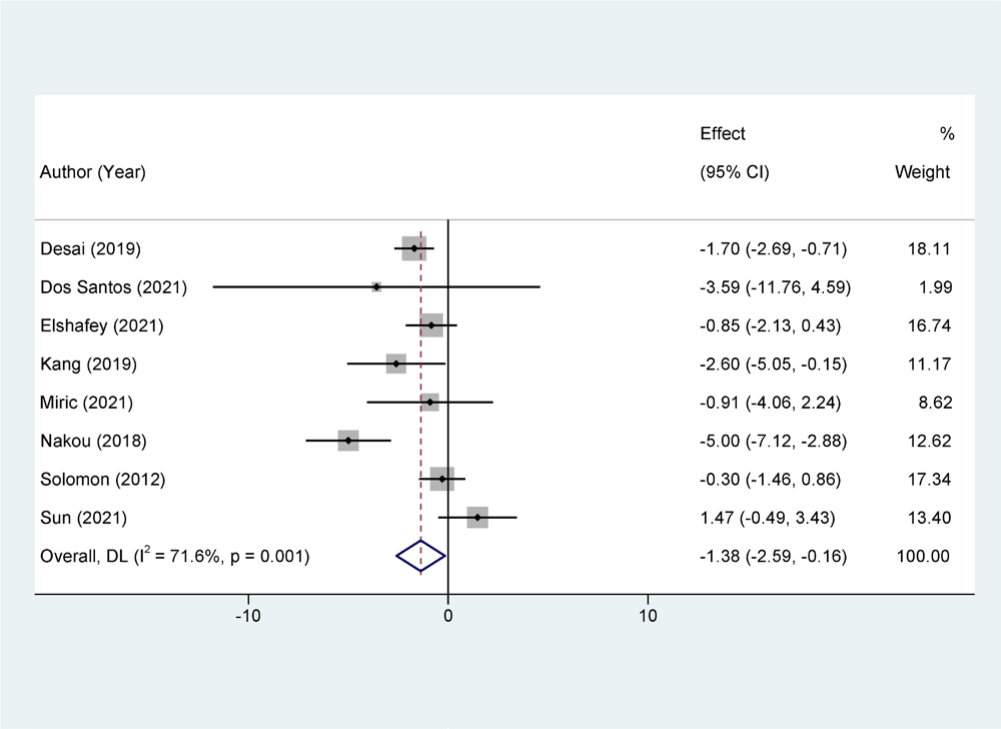
Forest plots of the impact of Sacubitril/valsartan on early mitral inflow velocity to early diastolic mitral annular velocity ratio.

A total of 8 studies reported data on the ratio of the early mitral inflow velocity to the early diastolic mitral annular velocity (E/e’ ratio), a key echocardiographic parameter for assessing diastolic function. Heterogeneity across studies was substantial, as evidenced by an *I*^2^ value of 71.6% and a *P* value of .001. Consequently, a random-effects model was employed for meta-analytic synthesis. The meta-analysis revealed that treatment with Sacubitril/Valsartan led to a significantly greater reduction in the E/e’ ratio when compared to the control group. Specifically, this treatment effect was highly statistically significant, with a mean difference (MD) of −1.38 and a 95% Confidence Interval (CI) ranging from −2.59 to −0.16 (*P* < .01). To provide a more nuanced understanding, it is worth noting that the E/e’ ratio is a pivotal metric for evaluating left ventricular filling pressures and, by extension, diastolic dysfunction—a condition often coexistent in HF patients (Fig. 3).

### 3.5. Meta-analysis on impact of Sacubitril/valsartan on left atrial volume index

**Figure 4.**
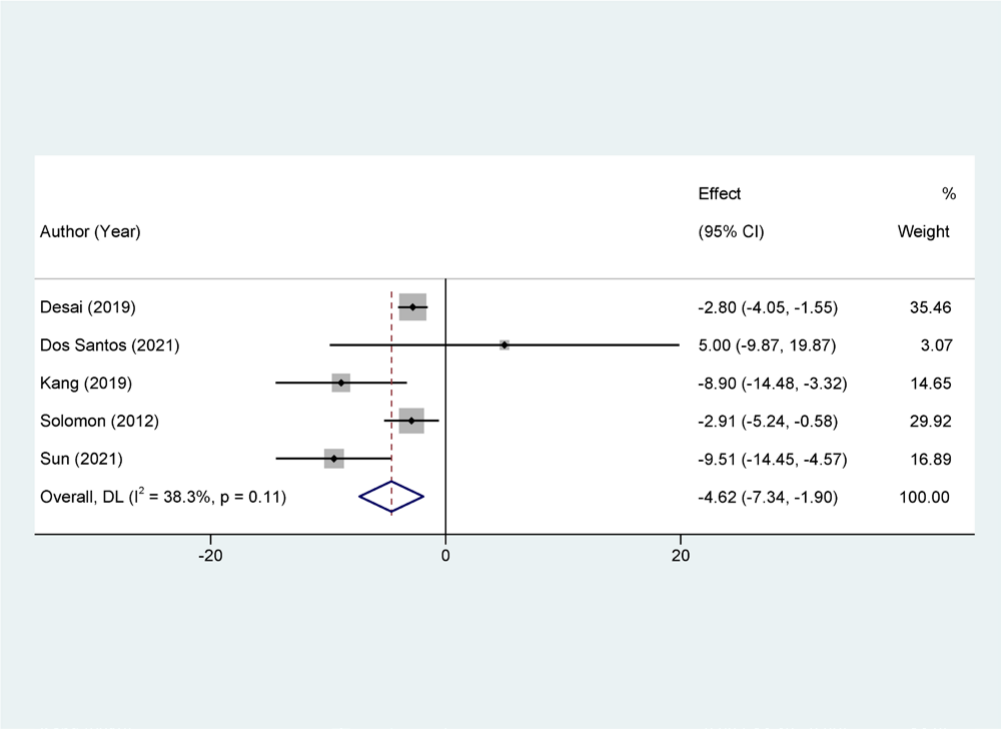
Forest plots of the impact of Sacubitril/valsartan on left atrial volume index.

Five studies were incorporated into the meta-analysis focusing on LAVi, an echocardiographic parameter commonly utilized for evaluating left atrial size and function. The heterogeneity among these studies was minimal, as reflected by an *I*^2^ value of 38.3% and a *P* value of .11. Given the low heterogeneity, a fixed-effects model was chosen for the meta-analytic synthesis. Our analysis revealed that treatment with Sacubitril/Valsartan resulted in a significant reduction in LAVi compared to the control group. The MD was −4.62, with a 95% CI ranging from −7.34 to −1.90 (*P* < .01) (Fig. 4).

### 3.6. Meta-analysis on impact of Sacubitril/valsartan on E/A ratio

**Figure 5.**
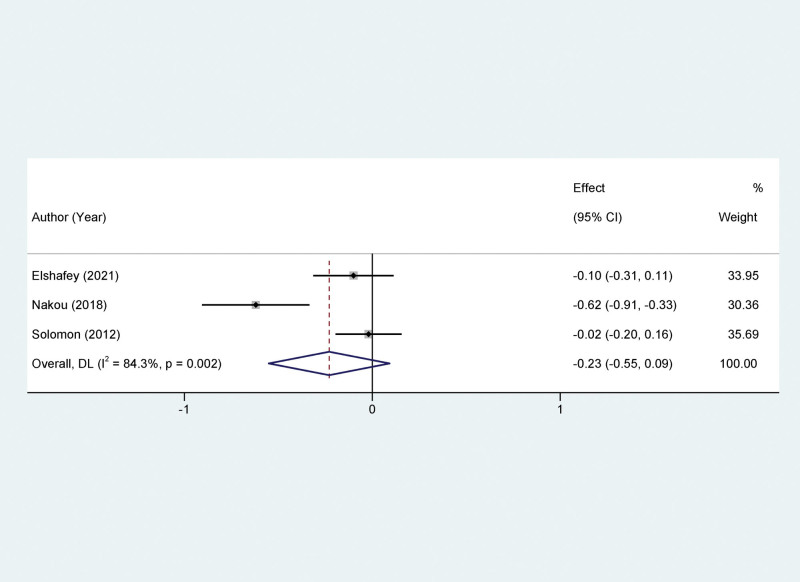
Forest plots of the impact of Sacubitril/valsartan on E/A ratio. E/A = ratio of early to late mitral inflow peak velocities.

Three studies were included in our meta-analysis to assess the impact of Sacubitril/Valsartan treatment on the E/A ratio, a Doppler-derived index frequently used to evaluate diastolic function. The studies exhibited substantial heterogeneity, as evidenced by an *I*^2^ value of 84.3%. Consequently, a random-effects model was employed for the analysis to accommodate this variability. The pooled results indicated that the treatment with Sacubitril/Valsartan did not yield a statistically significant improvement in the E/A ratio when compared to the control group. The MD was -0.23, within a 95% CI of −0.55 to 0.09 (*P* > .05, Fig. 5).

### 3.7. Sensitivity analysis on impact of Sacubitril/valsartan on early mitral inflow velocity to early diastolic mitral annular velocity ratio

**Figure 6.**
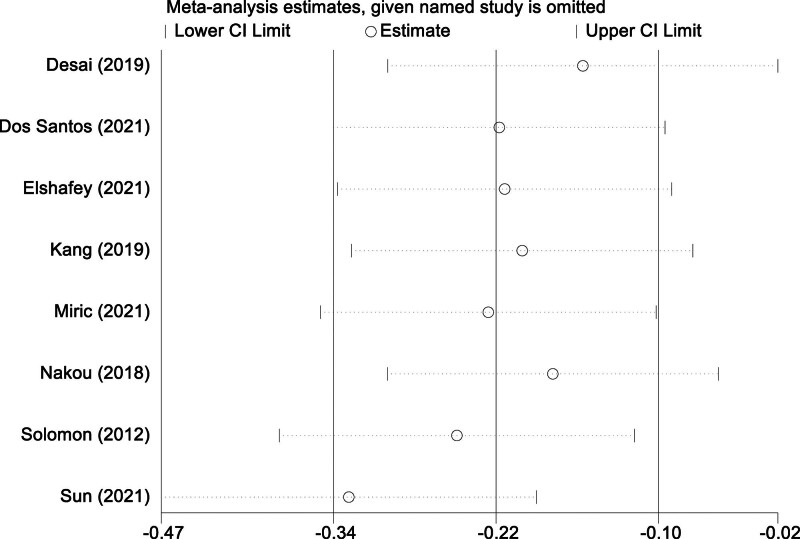
Sensitivity analysis on impact of Sacubitril/valsartan on early mitral inflow velocity to early diastolic mitral annular velocity ratio.

In light of the considerable heterogeneity detected among the studies incorporated into the meta-analysis, we executed a sensitivity analysis to gauge the robustness and dependability of the amalgamated findings. Through a meticulous procedure, each study was individually removed, and the aggregate effect sizes were recalculated with the remaining investigations. This exhaustive sensitivity exercise affirmed that the synthesized outcomes were neither disproportionately affected by any single study nor did their stability waver upon the removal of individual studies. Such resilience in the results across varying configurations attests to the robust nature of our primary conclusions. It also bolsters the trustworthiness of the aggregate findings yielded by this meta-analysis, as depicted in Figure 6.

### 3.8. Publication bias

**Figure 7.**
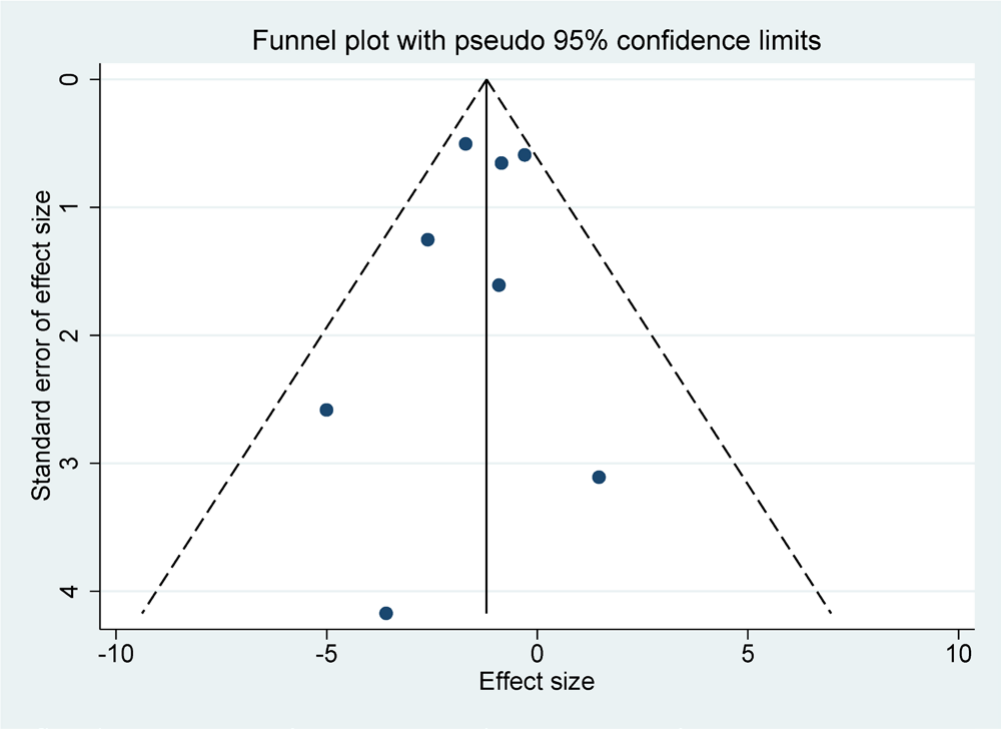
Funnel plot for publication bias.

The visual inspection of funnel plots generated from the incorporated studies demonstrated symmetrical distribution, suggesting an absence of notable publication bias (Fig. 7). Furthermore, Egger linear regression analysis substantiated the lack of statistically significant publication bias across various analytical conditions (*P* > .05 for all variables). These findings further corroborate the integrity and robustness of the results obtained from this meta-analysis.

## 4. Discussion

Heart failure (HF) continues to pose a critical medical burden globally, marked by elevated rates of morbidity, mortality, and substantial economic impact. Traditional treatment modalities, though beneficial in managing symptomatic relief, have proven insufficient in enhancing long-term outcomes for patients.^[18]^ Sacubitril/Valsartan, a novel angiotensin receptor-neprilysin inhibitor, made a marked entrance into the therapeutic landscape for HF. Initially documented by Solomon et al in 2012,^[16]^ its role in ameliorating hemodynamic parameters and lowering hospitalization rates was well-substantiated. Yet, there exist gaps in comprehensively understanding its influence on diverse cardiac metrics, particularly those related to diastolic function, an area increasingly explored through tissue Doppler imaging techniques.

Our meta-analysis methodically scrutinizes a multitude of diastolic parameters, including the E/A ratio, LAVi, and the ratio of early mitral inflow velocity to early diastolic mitral annular velocity (E/e’), to delineate the therapeutic impacts of Sacubitril/Valsartan on diastolic function in HF patients. Our findings reveal statistically significant improvements not only in LAVi but also in the E/e’ ratio, underscoring Sacubitril/Valsartan comprehensive role in enhancing cardiac hemodynamics. The reduction in the E/e’ ratio, a critical echocardiographic marker for assessing left ventricular filling pressures, underscores the potential of Sacubitril/Valsartan to transcend mere symptomatic relief, suggesting its capability to fundamentally improve cardiac function. Moreover, the significant decrease in LAVi elucidates the drug beneficial effects on left atrial size and function, directly correlating with a reduced cardiovascular risk profile, including a potential decrease in the incidence of atrial fibrillation. These insights bolster the evidence for Sacubitril/Valsartan multifaceted benefits, advocating its pivotal role in the nuanced management of heart failure, particularly for patients with diastolic dysfunction.

Our study reports statistically significant improvements in LAVi subsequent to Sacubitril/Valsartan therapy, thus affirming its salutary impact on left atrial function.^[19]^ Further enhancing the rigor of this analysis is our thorough sensitivity tests and evaluations for publication bias. This rigorous methodological underpinning elevates the reliability and validity of our findings, corroborating that Sacubitril/Valsartan has a more pronounced effect in mitigating diastolic dysfunction in HF patients compared to traditional ACEI/ARB medications.^[20]^

Recent animal and in vitro cellular studies suggest that Sacubitril, a component of Sacubitril/Valsartan, principally improves cardiac diastolic function through inhibition of neprilysin.^[21]^ This leads to reduced degradation of vasodilatory peptides including atrial natriuretic peptide, B-type, and C-type Natriuretic Peptides. Clarkson et al^[22]^ demonstrated that atrial natriuretic peptide infusion could increase myocardial diastolic rate and ameliorate cardiac diastolic function, possibly linked to elevated levels of cyclic guanosine monophosphate (cGMP) in the cardiac microcirculation. As an essential second messenger, cGMP can further modulate intracellular calcium transport and myofilament phosphorylation through the cGMP-protein kinase G signaling pathway.^[23,24]^ Moreover, animal studies indicate that Sacubitril/Valsartan can reduce left ventricular collagen expression levels and degrees of fibrosis in rats with both HFrEF and HFpEF, thereby decreasing the passive stiffness of the heart.^[25]^ Zile et al^[26]^ confirmed in a chronic HF patient Randomized Control Trial that treatment with Sacubitril/Valsartan significantly reduced biomarkers associated with pro-fibrotic signaling and collagen degradation.

We rigorously evaluated the risk of bias in the included studies using the Cochrane Collaboration Risk of Bias assessment tool (version ROB 1.0), categorizing each domain as low, unclear, or high risk of bias. This systematic assessment was pivotal in interpreting the robustness of our findings. Specifically, we found that while half of the studies exhibited low susceptibility to bias across all evaluative dimensions, indicating robust methodological quality, the presence of high or unclear risks in critical domains such as blinding of participants and outcome reporting in the remaining studies necessitated a cautious approach in the interpretation of our results. The heterogeneity introduced by the varying levels of risk across studies prompted us to employ both fixed-effects and random-effects models based on the *I*^2^ statistic to accurately reflect the diversity in study quality. Studies with a high risk of bias, particularly in the domains of blinding and selective outcome reporting, were scrutinized for their potential to overestimate the effect sizes.

The current meta-analysis has several limitations. First, the study relies on data from a relatively small number of included trials, thereby potentially introducing selection bias and reducing the generalizability of the findings. Second, we did not have access to individual patient-level data, which restricts our ability to conduct more detailed subgroup analyses or meta-regression. Third, heterogeneity among studies in terms of patient characteristics, study designs, and methods for assessing diastolic function could introduce confounding factors that might have influenced the results. Fourth, most of the included studies were of short to medium duration, limiting the assessment of long-term outcomes related to diastolic function. Lastly, the meta-analysis did not consider the potential impact of other medications or comorbid conditions on the observed treatment effects.

Furthermore, the majority of HF cases included in our analysis presented with left ventricular dysfunction, raising the possibility that observed improvements in diastolic function may partially derive from enhancements in ejection fraction (EF). Given that EF improvement can contribute to overall cardiac function enhancement, including diastolic function, there is a potential risk of overestimating the specific impact of Sacubitril/Valsartan on diastolic improvements without adequately accounting for this aspect. This underscores the necessity for future research to meticulously dissect the relationship between EF improvement and diastolic function amelioration, and to investigate whether the effects of Sacubitril/Valsartan vary among HF patients with differing levels of EF. Acknowledging this limitation not only enhances the transparency and completeness of our study but also sets a direction for subsequent inquiries into the multifaceted benefits of Sacubitril/Valsartan in the nuanced management of HF.

## 5. Conclusions

In summary, our study reveals that Sacubitril/Valsartan significantly improves diastolic indicators such as E/e′ and LAVi in patients with HF. While no statistical difference was observed in E/A ratios between the treatment and control groups, the overall evidence suggests that Sacubitril/Valsartan is more effective than conventional HF medications in enhancing diastolic function.

## Author contributions

**Data curation:** Yanbin Song.

**Formal analysis:** Jinfu Li, Yanbin Song.

**Methodology:** Jinfu Li, Yanbin Song.

**Supervision:** Fengyun Chen.

**Visualization:** Fengyun Chen.

**Writing – original draft:** Jinfu Li.

**Writing – review & editing:** Fengyun Chen.
